# Volumetric Evaluation of Pharyngeal Airway after Functional Therapy

**DOI:** 10.1155/2021/6694992

**Published:** 2021-02-18

**Authors:** Ersin Yıldırım, Şeniz Karaçay

**Affiliations:** Health Sciences University, Faculty of Dentistry, Department of Orthodontics, Istanbul, Turkey

## Abstract

The aim of this study was to evaluate three-dimensional (3D) effects of Twin-block functional appliance (TB) on the pharyngeal airway by using cone beam computed tomography (CBCT). A total of 30 patients (14 females, 16 males; mean age 12.50 ± 1.23 and 12.83 ± 1.17 years, respectively) with skeletal Class II malocclusion were included in this study and were treated with TB. On the pretreatment (T1) and posttreatment (T2) CBCT scans, volumetric changes in the pharyngeal airway; SNA, SNB, and ANB angles; and bilateral effective mandibular (Co-Gn) and midfacial length (Co-A) were also evaluated. The statistical differences were accessed by Wilcoxon signed-rank tests, and Mann-Whitney *U* tests were used to analyze the scores of male and female subjects. In this study, an increase was observed in SNB and Co-Gn (*p* < 0.01) while a decrease in ANB and SNA (*p* < 0.01 and *p* < 0.05, respectively) was found. However, increase in midfacial length was not statistically significant (*p* > 0.05). In the evaluation of volumetric pharyngeal airway changes, statistically significant increases (*p* < 0.01) in the upper and lower division and total airway volume were determined. Gender differences were insignificant for all measurements (*p* > 0.05). Volumetric changes in the pharyngeal airway after functional therapy can be successfully evaluated by CBCT images. The anterior repositioning of the mandible by TB increases the mandibular length and pharyngeal airway volume in patients with retrognathic mandible.

## 1. Introduction

The upper airway has been the subject of interest in orthodontic literature since its obstruction may affect the development of dentofacial structures by altering the breathing pattern of growing patients [1]. Chronic mouth breathing has been reported as an etiologic factor of maxillary arch deficiency, crossbite, posterior rotation of the mandible, mandibular retrognathia, and long face pattern [2]. Correlation between airway dimensions and different malocclusions has been investigated to evaluate the relationship between development of the dentofacial complex and nasopharyngeal and oropharyngeal structures [3–5]. When the Angle skeletal classification was considered, negative correlation between oropharynx volume and ANB angle was reported [6–9]. El and Palamo [8] reported a positive correlation between SNB and oropharyngeal volume. Trenouth and Timms [7] showed that oropharyngeal volume is correlated positively with the mandibular length, third cervical vertebra and hyoid bone distance, and the cranial base angle. These studies revealed that Class II patients had significantly reduced airway volume according to patients with Class I and Class III malocclusion [8–11]. As a result, respiratory function problems such as upper respiratory resistance syndrome, snoring, and obstructive sleep apnea (OSA) can be observed more frequently in Class II patients due to retrognathic mandible [12, 13].

Growing children with retrognathic mandible have been treated with various removable or fixed functional appliances for many decades. Twin-block (TB) is one of the most frequently used removable functional appliances. This appliance was developed by Clark and comprises of separate maxillary and mandibular bite block units with occlusal inclined planes that allow the mandible to be displaced downward and forward on closure [14]. Its popularity depends on high patient cooperation and its ability to produce rapid correction of sagittal discrepancy due to full-time wear. In some previous studies, the effects of TB on the skeletal and dentoalveolar structures [15–19] and temporomandibular joint [20] were evaluated. Additionally, Zhang et al. [21] and Shete and Bhad [22] reported that TB appliance may improve the obstructive sleep apnea (OSA) symptoms in patients with mandibular retrognathia. Generally, the pharyngeal airway dimension changes following functional therapy were examined through two-dimensional (2D) lateral cephalograms [23–25] or 2D cephalometry extracted from cone beam computed tomography (CBCT) [26, 27]. However, because of its 2D nature and inability to provide volumetric data, the value of lateral cephalograms is limited in the studies concerning the airway.

Literature review revealed two recent studies in which the upper airway was examined three-dimensionally following treatment with TB. In one of the determined studies, the effects of TB were compared with Forsus Fatigue Resistance Device (FFRD) in patients with skeletal Class II malocclusion [28]. Other study evaluated volumetric alterations of the upper airway retrospectively in growing patients who were treated with TB [29]. However, in both recent studies, the initial results were compared with CBCT values taken after active orthodontic treatment. 3D volumetric changes in the pharyngeal airway achieved with TB treatment need to be clearly identified. This study was designed to evaluate the immediate treatment effects of TB on the pharyngeal airway volume. To our knowledge, there is no other study evaluating the 3D effects of TB on the pharyngeal airway independent from the effects of fixed orthodontic approaches following the functional therapy.

## 2. Materials and Methods

### 2.1. Subjects

Ethical approval of this prospective study was obtained from the Ethics Committee of Gulhane Military Medical Academy in Ankara, Turkey (1491-107-11/1539-1588). A total of 30 patients (14 females, 16 males; mean age 12.50 ± 1.23 and 12.83 ± 1.17 years, respectively) participated in this study. Informed consent forms were taken from all patients.

Patient selection criteria were as follows:

- Class II skeletal relation with retrognathic mandible and normal maxilla (ANB > 4°)
- Class II dental relation bilaterally (molar and canine) and minimum or no crowding
- At least 5 mm overjet
- Normal or horizontal growth pattern
- Significant growth potential (in stages 3–4 according to the cervical vertebrae maturation indicators (CVMI) [30])

Exclusion criteria were as follows:

- Respiratory disorders and/or OSA
- Nasooropharyngeal obstructions
- History of upper airway surgeries like adenoidectomy or tonsillectomy
- Syndromes and craniofacial abnormalities

All patients were treated with TB, and the mean treatment time was 7.4 months. On the pretreatment (T1) and posttreatment (T2) CBCT scans, skeletal alterations and volumetric changes in the pharyngeal airway were evaluated.

### 2.2. Data Collection and Measurements

Pre- and posttreatment images were obtained using the ILUMA (IMTEC, 3M Company, Ardmore, Oklahoma, USA) CBCT machine while the patients were in an upright sitting posture. The rest position of the tongue and maximum intercuspation were also required during the scanning process. The scanning parameter of the CBCT was 120 kV and 3.8 mA. The scan was made in 20 seconds, a single 360° rotation with a 21.1 × 14.2 cm field of view. Slice thickness and isotropic voxel size were 0.290 mm. The CBCT images were exported into Digital Imaging and Communications in Medicine (DICOM) file format. Image reconstruction, landmark identifications, and measurements were made using SimPlant Master Crystal v13 (Materialise Dental, Leuven, Belgium) software.

**Figure 1:**
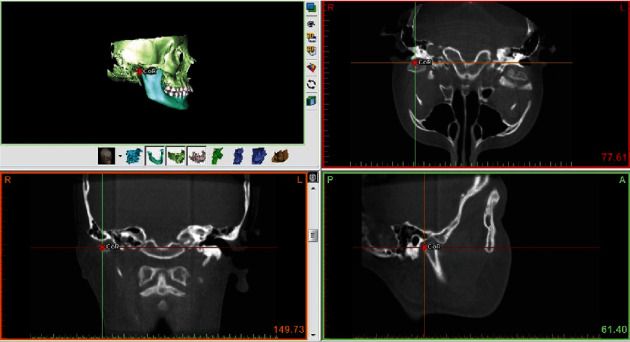
Localization of landmarks was controlled in all dimensions on the reconstructed 3D models.

On T1 and T2 images, skeletal alterations were evaluated by angular (SNA, SNB, ANB) and linear (Co-A, Co-Gn bilaterally) 3D cephalometric measurements. Localization of cephalometric landmarks was carried out by controlling in all dimensions of the reconstructed 3D images (Figure 1).

**Figure 2:**
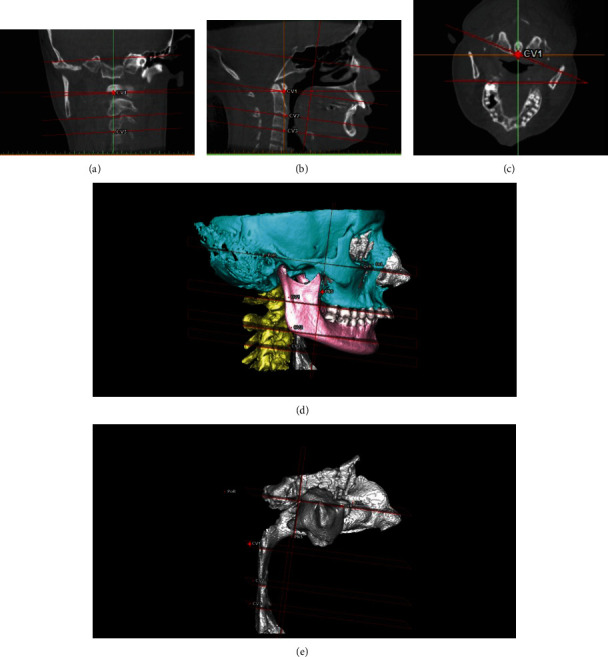
Reference planes generated on the 3D images to evaluate airway volume.

To assess volumetric changes, the pharyngeal airway was segmented on T1 and T2 images with a constant thresholding values (min.:-1024, max.:-1000 Hounsfield units) to establish uniform segmentation. Five reference planes were generated on the segmented 3D images (Figure 2):

- FH (Frankfort Horizontal) plane: left and right orbitale (Or) and right portion (Po) are the three points defining this plane
- CV1 plane: a plane trough passing in the most caudal medial point of the first cervical vertebra and parallel to the FH plane
- CV2 plane: a plane trough passing in the most caudal medial point of the second cervical vertebra and parallel to the FH plane
- CV3 plane: a plane trough passing in the most caudal medial point of the third cervical vertebra and parallel to the FH plane
- PNS plane: a plane trough passing in the posterior nasal spine (PNS) and perpendicular to the FH plane

For the pharyngeal airway measurement, the following anatomical structures and planes are used as borders: the PNS plane as the anterior border, the most superior border as the upper border, posterior and lateral walls of the pharynx as the posterior and lateral borders, and the CV3 plane as the lower border.

**Figure 3:**
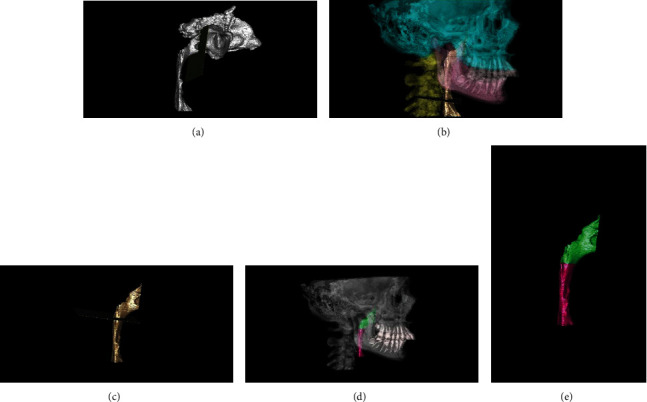
Segmentation of superior (green area) and inferior airway (red area) compartments.

To cut 3D images from the anterior and inferior borders, the software's “custom planar osteotomy” function was used to create a plane of 0.1 mm thickness based on the PNS plane and CV3 plane (Figures 3(a) and 3(b)). By using the same method, the 3D images were also cut from the CV1 plane to divide the total airway into two parts as superior and inferior airway compartments (Figures 3(c)–3(e)). Airway volume was calculated in cubic millimeter. This process was applied on T1 and T2 images separately. Landmark identifications and measurements were made by the same author (E.Y.).

### 2.3. Statistics

A power analysis established by G∗Power software (v3.1.3; Franz Faul, Universität Kiel, Germany) revealed that the sample size of 30 patients provided more than 80% power to detect significant differences with an effect size of 0.50 between the two measurements at a 0.05 significance level. The measurements of 10 patients were repeated 1 month later.

Statistical analyses were performed using the Gnu PSPP software (Free Software Foundation, http://www.gnu.org/software/pspp/get.html). The Kolmogorov-Smirnov normality test (with Lilliefors significance correction) and Levene's variance homogeneity test were applied to the data. The mean measurement values and standard deviations (SD) were calculated for T1 and T2. The Wilcoxon signed-rank test was used to compare T1 and T2 scores, and Mann-Whitney *U* tests was used to compare the scores of male and female participants.

## 3. Results

Reliability was evaluated by using intraclass correlation coefficients (ICCs) and Bland-Altman plots (Table 1). The measurements showed excellent intraexaminer repeatability. Means and standard deviations, comparison of volumetric airway dimensions, and skeletal angular and linear measurements for T1 and T2 are given in Table 2.

Overjet was reduced, and Class I molar and canine relationship was obtained at the end of TB therapy. When the skeletal alterations were evaluated, increase in SNB (*p* < 0.01) and decrease in SNA (*p* < 0.05) were determined. ANB decreased significantly (*p* < 0.01) depending on these alterations. These measurements revealed that mild repositioning of the maxilla in the posterior direction and significant repositioning of the mandible in the anterior direction caused a reduction in ANB. Effective mandibular length (Co-Gn) increased significantly for both right and left sides (*p* < 0.01) but increase in effective midfacial length (Co-A) was not significant at either side (*p* > 0.05).

In the evaluation of volumetric pharyngeal airway changes, statistically significant increases (*p* < 0.01) in the upper and lower division and total airway volume were determined. Gender differences were insignificant for all measurements (*p* > 0.05).

## 4. Discussion

Functional therapy is the preferred treatment choice in growing patients if the skeletal Class II malocclusion depends on retrognathic mandible. Functional appliances are also effective in OSA by increasing the posterior airway space [21, 22]. In orthodontic literature, Ozbek et al. [12] were the first who evaluated the effects of functional therapy on the oropharyngeal airway in patients with skeletal Class II morphology due to deficient mandible. They revealed that oropharyngeal airway dimensions increased after Harvold-type activator treatment. Restrepo et al. [31] investigated the same issue with prepubertal patients treated with Bionator or Klammt appliance. They found increase in the area of the adenoid tissue and concluded that mandibular advancement before the growth peak could be a solution for airway obstructions. Yassaei et al. [32], also reported that Faramand functional appliance increased pharyngeal airway dimensions and caused positional changes of tongue and hyoid bone. Jena et al. [24] reported that TB and Mandibular Protraction Appliance-IV were both effective in improving pharyngeal airway space, but the improvement with TB was greater significantly. Vinoth et al. [23] also determined a significant increase in the upper and lower pharyngeal width and the bony nasopharyngeal area with TB therapy. Kinzinger et al. [33] investigated the influence of two fixed functional appliances (Herbst and Functional Mandibular Advancer) on the posterior airway space (PAS) morphology. They concluded that two appliances had the same effects on PAS but were not able to prevent OSA. Similarly, Ozdemir et al. [34] did not find any significant changes on PAS after treatment with FFRD fixed functional appliance.

In the studies mentioned above, effects of functional therapy on pharyngeal dimensions were assessed by lateral cephalometric measurements but lateral cephalograms are only available for 2D evaluation. Because they are 2D projection images of a 3D structure, cross-sectional and volumetric assessment is not possible with lateral cephalograms. Kinzinger et al. [33] reported that the results of their study were not absolutely reliable because there was not enough information about the volume of the PAS. Guijarro-Martinez and Swennen [35] concluded that 3D evaluation of the airway with CBCT provides accurate and reliable results. In our study, CBCT images were used to assess 3D changes in the pharyngeal airway volume. CBCT uses significantly lower radiation dose according to the medical CT, and in some studies, it has been reported to have radiation dose equivalent to traditional imaging methods such as full mouth radiographic series [36]. We did not take cephalometric radiographs to reduce the exposed radiation dose, and cephalometric analyses were also made on CBCT images.

Alhammadi et al. [28] investigated skeletal and pharyngeal airway changes in skeletal Class II female patients treated with two different functional appliances (TB and FFRD) compared with untreated controls three-dimensionally on CBCT images. However, there is no information about active treatment period or pre- and posttreatment CBCT image acquisition times. Li et al. [29] also used CBCT to evaluate the morphological changes of the upper airway in growing patients treated with TB. In this study, not only the statistical differences between pre- and posttreatment results of the TB group but also the posttreatment and untreated control data were compared. However, CBCT scans of the control group were collected only before orthodontic therapy. Orthodontic treatment period of the TB group was 13.6 months; in other words, there was 13.6 months between pre- and posttreatment data of the TB group. Valuable findings might be obtained by comparing the difference between pre- and posttreatment results of the TB group with the difference that was obtained from untreated Class II patients two times within an approximately 13-month period. In our opinion, since control data was taken only at the initial phase of the study, comparing the posttreatment TB results with the initial data of the control group is not scientifically reasonable.

In the present study, we aimed to identify the immediate treatment effects of TB on the volume of the pharyngeal airway independent from the effects of any other orthodontic fixed treatment approaches. The average TB therapy in our study was 7.4 months. There was no control group in this study so we could not compare the results with untreated skeletal Class II patients. Absence of the control group may be perceived as the disadvantage of our study. However, in a growth study examining pharyngeal airway change, Mislik et al. [37] reported that PAS dimensions in growing children are being established during early childhood and remained unchanged between ages 6 and 17 years. From this point of view, we did not need to construct a nontreated control group in our study. Considering the short duration of the study (7.4 months), it can be concluded that the changes that were examined in the PAS dimensions were independent from the natural growth of the patients.

In our study, SNB increased and ANB decreased after TB therapy. Class II sagittal relation improved significantly due to the forward mandibular displacement and increase in the effective mandibular length (Co-Gn). Similar to our results, several clinical studies investigating changes caused by the TB have demonstrated its effectiveness in increasing mandibular growth [15–21, 23, 24, 28, 29]. Although a mild decrease was found in SNA angle, midfacial length (Co-A) did not change revealing that TB has only restraining effect on the maxillary growth. The results related with maxilla were in accordance with several previous studies [16–19, 23, 28].

Increase in the superior and inferior compartments of the airway was found in our study. These increases also led to a significant increase in total airway volume. The studies of Vinoth et al. [23], Jena et al. [24], and Li et al. [29] also showed the effectiveness of TB in the improvement of the PAS dimensions. Similar results were also reported by several investigators that studied with different removable functional appliances like Harvold-type activator [12], Klammt or Bionator [31], and Faramand functional appliance [32]. Our results are also in accordance with Schütz et al. [38], who reported a significant increase in the PAS dimension with maxillary expansion and Herbst therapy. On the other hand, conflicting with our results, Kinzinger et al. [33] and Ozdemir et al. [34] who investigated the effects of a fixed functional appliance could not find significant alterations in the morphology of PAS. The location of the mandible with respect to the anterior cranial base affects the pharyngeal airway volume. The space between the mandibular corpus and the cervical column is reduced due to the retrognathic mandible and leads to a posterior postural position of the tongue and soft palate [6, 39]. In our study, pharyngeal volume increased due to the forward mandibular displacement and increase in the mandibular length. Schütz et al. [38] and Li et al. [29] also concluded that functional therapy caused forward position of the tongue and increased PAS by displacing the mandible and hyoid bone forward. In the study of Ozdemir et al. [34], FFRD did not cause any significant changes in PAS because there was no statistically significant correction in Class II malocclusion. ANB angle did not change, and reduction of overjet was caused by dentoalveolar alterations in their study. Tongue area and intermaxillary space area increased because of these dentoalveolar changes. However, these changes were not enough to affect the position of tongue and hyoid bone and subsequently the dimension of PAS. Alhammadi et al. [28] also showed significant increase in PAS in the TB group compared to both the FFRD and natural growth. Findings of our study revealed that PAS may increase in Class II patients with retrognathic mandible if forward mandibular displacement can be obtained by functional therapy. In this situation, removable functional appliances may be more efficient to increase PAS according to fixed appliances since dentoalveolar camouflage is more in fixed functional appliances rather than advancement of the mandible [28, 34, 40].

Hence, TB treatment may prevent OSA and other respiratory problems in the future by providing an increase in the pharyngeal airway volume while providing the skeletal correction of Class II malocclusions by directing the mandibular growth. The present study was designed to evaluate the immediate treatment effects of TB on pharyngeal airway dimensions. In the future, a study including 3D airway analysis with long-term implication of treatment is recommended.

## 5. Conclusion

- CBCT is an efficient technique to evaluate the volumetric changes in the pharyngeal airway after functional therapy
- TB therapy increases the effective mandibular length and leads to anterior repositioning of the mandible
- Functional treatment with TB increases the pharyngeal airway volume in patients with retrognathic mandible

## Figures and Tables

**Table 1:** Intraclass correlation coefficients (ICCs) and Bland-Altman plots of the measurements.

Measurements	Intraclass correlation coefficients		Bland-Altman analysis	
	ICCs	95% CI	Bias	95% CI
Superior airway volume (mm^3^)	0.899	0.872 to 0.962	28.931	-115.702 to 173.564
Inferior airway volume (mm^3^)	0.848	0.782 to 0.889	13.835	-137.703 to 165.373
Total airway volume (mm^3^)	0.876	0.758 to 0.945	42.765	-161.578 to 247.108
SNA (°)	0.862	0.540 to 0.964	0.023	-0.735 to 0.781
SNB (°)	0.835	0.469 to 0.956	0.025	-0.668 to 0.718
ANB (°)	0.987	0.947 to 0.997	-0.029	-0.149 to 0.091
Co_L_-A (mm)	0.943	0.790 to 0.986	0.201	-0.959 to 1.361
Co_R_-A (mm)	0.970	0.883 to 0.992	0.301	-0.589 to 1.191
Co_L_-Gn (mm)	0.957	0.837 to 0.989	-0.063	-1.079 to 0.953
Co_R_-Gn (mm)	0.945	0.797 to 0.986	0.058	-1.021 to 1.137

CI: confidence interval; Co_L_: left condylion; Co_R_: right condylion.

**Table 2:** Descriptive statistics of the measurements with results of Wilcoxon signed-rank tests.

Measurements	T1		T2		*p*
	Mean	SD	Mean	SD	
Superior airway volume (mm^3^)	11639.84	3855.28	14404.47	6190.05	0.007
Inferior airway volume (mm^3^)	9958.84	4752.42	14858.57	6494.36	0.005
Total airway volume (mm^3^)	21598.68	7395.58	29263.04	10876.60	0.005
SNA (°)	81.65	1.61	81.33	1.48	0.028
SNB (°)	74.63	1.44	78.36	1.53	0.005
ANB (°)	7.02	0.96	2.97	0.79	0.005
Co_L_-A (mm)	96.33	5.07	98.84	4.09	0.285
Co_R_-A (mm)	97.86	5.27	99.43	4.66	0.508
Co_L_-Gn (mm)	114.61	4.85	118.60	4.76	0.005
Co_R_-Gn (mm)	116.04	4.64	119.49	5.12	0.005

SD: standard deviation; Co_L_: left condylion; Co_R_: right condylion.

## Data Availability

The data used to support the findings of this study are included within the article.
